# Influence of the Alkali Treatment of Flax and Hemp Fibers on the Properties of PHBV Based Biocomposites

**DOI:** 10.3390/polym13121965

**Published:** 2021-06-14

**Authors:** Wiesław Frącz, Grzegorz Janowski, Łukasz Bąk

**Affiliations:** Department of Materials Forming and Processing, Rzeszow University of Technology, 35-959 Rzeszow, Poland; gjan@prz.edu.pl (G.J.); lbak@prz.edu.pl (Ł.B.)

**Keywords:** poly (3-hydroxybutyric-co-3-hydroxyvaleric acid) (PHBV), flax, hemp, short fibers, injection molding, extrusion, properties

## Abstract

This study assessed the impact of alkali treatment of hemp and flax fibers on mechanical properties (determined by means of the uniaxial tensile test, impact tensile strength test and hardness test), processing properties (the course of the extrusion and injection process) and usable properties (shrinkage of molded pieces, degree of water absorption) of biocomposites on the base of poly (3-hydroxybutyric-co-3-hydroxyvaleric acid) (PHBV) biopolymer. For this purpose, 1 mm of length flax and hemp fibers was surface-modified by means of aqueous solution of NaOH (sodium hydroxide) with concentrations of 2%, 5% and 10%. The composites were made using the extrusion technology. The test specimens were produced by injection molding technology. In total, eight types of biocomposites with modified and non-modified fibers were produced, and each biocomposite contained the same filler content (15 wt.%). Their properties were compared in some cases with pure PHBV polymer. In the case of biocomposites filled with hemp fibers, it was noted that an increase of the alkalizing solution concentration improved most of the tested properties of the obtained biocomposites. On the other hand, in the case of flax fibers, there was a significant decrease in most of the mechanical properties tested for the composite containing fibers etched by 10% NaOH solution. The obtained results were verified by examining fibers and the destroyed specimens with a scanning electron microscope (SEM) and an optical microscope, which confirmed, especially, the significant geometry changes of the flax fibers etched by 10% NaOH solution. This procedure also resulted in a significant change of processing properties—a composite of this fiber type required about 20 °C lower temperature during the extrusion and injection molding process in order to obtain the right product. These results lead to the important conclusion that for each filler of the plant-origin and polymer matrix, the fiber alkalization method should be selected individually in order to improve the specific properties of biocomposites.

## 1. Introduction

The main components of plant-origin fibers are cellulose, hemicellulose and lignin. Cellulose is synthesized in plants, trees and grasses and even in some varieties of algae, fungi and bacteria. The most common and abundantly available cellulose is contained in cotton (about 90% cellulose content) [1,2]. The proportion of cellulose in plants may vary depending on the species and age of the plant. Cellulose is a hydrophilic biopolymer consisting of a linear chain of glucose molecules linked by β-1.4-glycosidic bonds that contain hydroxyl groups. These groups form intermolecular and intramolecular hydrogen bonds with the macromolecule itself as well as with other cellulose macromolecules or polar molecules. Fibers of plant origin are hydrophilic. Although the chemical structure of cellulose from different natural fibers is the same, the degree of polymerization is different. The mechanical properties of the fibers largely depend on the degree of polymerization of the cellulose. In plants, cellulose has two different unit cells: I and II. In an alkaline environment, cellulose I is converted to II. Form II is more thermodynamically stable, which provides higher thermal resistance and strength [2]. Hemicellulose is a heterogeneous biopolymer of a group of polysaccharides and their derivatives, linked by β-glycosidic bonds to form branched chains. In contrast to alkaline-soluble cellulose, hemicellulose is less resistant to dilute acids [3]. Lignin fills the spaces between the polysaccharide fibers, binding them together. The presence of these component fibers causes a stiffening of the cell walls to protect against chemical and physical damage. Lignin is a polymer with monomers derived from phenolic alcohols. The presence of this compound in the fibers reduces the penetration of water into the cells and stiffens the material [4]. It should be noted that individual components of plant-origin fibers degrade in various temperature ranges. Above 200 °C, hemicellulose degrades, and above 300 °C, cellulose degrades. Lignin is the most thermally resistant—it degrades above 350 °C. Obviously, the mechanical properties of the fibers decrease with the degradation of successive compounds that build plant fiber [5].

Hydroxyl groups (OH) in cellulose, hemicellulose and lignin build a large number of hydrogen bonds inside the macromolecule and between macromolecules in the cell wall of plant-origin fibers. The action of water on the plant fibers causes these bonds to break. The hydroxyl groups then form new hydrogen bonds with the water molecules that promote the swelling of the fiber. The swelling of the cell wall generates very strong forces. The theoretical value of the pressure may be about 165 MPa [6], but the real swelling pressure is half of the calculated value [7,8]. Cellulose fibers interact with water not only on the surface but also in the entire volume. The structure of cellulosic materials consists of crystalline and amorphous regions. Amorphous areas easily absorb chemical compounds such as dyes and resins, and the presence of crystalline areas makes chemical penetration difficult [9].

The possibility of water absorption by cellulose fibers depends on the following aspects, among others [1,2]:

– Purity of cellulose: Raw cellulosic material, such as unwashed sisal fibers, absorbs at least twice as much water as washed fibers due to a 24% pectin content.
– Degree of crystallinity: All the OH groups in the amorphous phase are accessible to water, while only a small amount of water interacts with the surface OH groups of the crystalline phase.

The main disadvantage of cellulose fibers is their highly polar nature, which makes them incompatible with non-polar polymers. The poor resistance to water absorption makes the use of natural fibers less attractive for applications involving external factors such as rain, snow and hail [10].

The polymers have different affinity for the fiber due to the difference in their chemical structure [11,12,13,14]. Effective filling of composites with plant fibers depends on moisture content, fiber–matrix interfacial adhesion, cellulose content and the degree of crystallinity. In order to increase the possibility of adhesion of the fibers to the polymer matrix, reduce water absorption, increase the proportion of cellulose in the fiber and increase the degree of crystallinity, different methods of fiber surface modification are used.

Cellulose fibers can be physically modified, e.g., by calendering, stretching, thermal treatment, plasma treatment and corona discharge. In turn, chemical methods of modification of fibers include silanization, alkalization, acetylation, graft copolymerization and modification with enzymes [15,16]. One of the most popular methods of chemically modifying the surface of fibers is alkalization, often referred to as mercerization. This method is based on the action of a sodium hydroxide solution (at the appropriate concentration) on the plant fiber.

The reaction of sodium hydroxide with cellulose is shown by the equation below:

$$Cellulose\;chain-OH+NaOH\to \;Cellulose\;chain-O^{-}+Na^{+}+H_{2}O+other\;substances$$

Hydroxyl groups, which are polar in nature, are blocked by the action of sodium, and water is released as a byproduct of the reaction. Moreover, the abovementioned chemical treatment removes lignin, pectin, waxy substances and natural oils covering the outer surface of the fiber cell wall. This can increase the proportion of cellulose in the fiber and the roughness of the cell wall surface. Additionally, alkalization enables the conversion of type I cellulose to type II, which increases the thermodynamic resistance and strength properties of cellulose and consequently of the entire plant fiber [16,17,18,19]. It is worth noting that alkalization also shortens the cellulose chains [19].

The effect of surface modification, and in particular the alkalization of hemp and flax fibers, has been the subject of many studies [20,21,22,23,24,25,26,27]. Ouajai and Shanks [20] found that pectin and hemicellulose were removed during the alkalization from hemp fibers. Mwaikambo et al. [21] conducted FTIR studies to confirm that hemicellulose was removed by treatment with a base solution of cellulose fibers, including hemp fibers. In [22], the mercerization of hemp fiber increased the tensile strength and bending strength of PP–hemp fiber composites, which indicates an improvement in interfacial bonding after alkaline treatment. The highest values of tensile and bending strength of PP composites reinforced with hemp fibers were obtained with a 4% sodium base solution treatment. Meanwhile, the tensile and bending strength of the composite filled with fibers treated with 6% NaOH solution was lower than in the case of composites etched with 2% and 4% NaOH solutions. The results of the work by Hu et al. [23] also indicate that the tensile strength and flexural strength values are higher when the hemp fibers are alkalized. The authors also noted that the alkaline treatment of the fibers effectively removed non-cellulose fractions, which resulted in a 25.9% reduction in fiber weight and had a positive effect on the mechanical properties by improving the fiber–matrix bonds. The surface morphology of the fiber was significantly changed after the treatment by removing the non-cellulose layer on the fiber, resulting in a higher surface roughness. In the work of Pickering [24], the alkalization of hemp fibers improved their adhesion to polymer matrices. This was due to a change in the fiber surface properties, removal of non-crystalline components such as hemicellulose, lignin and pectin [25] and removal of waxes and fatty acids that may adversely affect interfacial bonds. Van de Weyenberg and co-authors [26] investigated the influence of some methods of physical modification of flax fibers on the properties of composites (fiber volume content was 40%) with a polyethylene matrix. Li and co-authors [27] investigated the properties of polyethylene-flax fiber biocomposites. The composites contained 10 wt.% fiber contents and were processed by extrusion and injection molding. Five surface modification methods were used: mercerization, silanization, potassium permanganate, acrylic acid and soda chlorite treatment to improve the bond between the fibers and the matrix. It was noticed that the tensile strength of the biocomposite increased after the flax fibers were alkalized.

An important problem is the constantly increasing amount of plastic waste in Europe and all over the world. It should be mentioned that these materials are mostly of petrochemical origin [28], are not biodegradable and are recycled with varying degrees of success. Poly (3-hydroxybutyric-co-3-hydroxyvaleric acid) (PHBV) belonging to the group of polyhydroxyalkanoates (PHAs) is a polymer of natural origin that is fully biodegradable. Due to its high production costs, it is rarely used as an injection molded material and therefore has little commercialization potential. One of the methods of reducing production costs and improving the properties of the abovementioned biopolymer is the use of plant-origin fibers as a filler in the PHBV matrix.

Quite a large number of studies conducted on PHBV composites with a matrix of plant fibers concern the possibility of processing mainly by compression molding of thin layers or films [29,30,31,32]. Some results have been obtained with composites manufactured on a very small scale [33]. Both compression molding of thin composite sheets and processing on mini extruders and mini injection molding machines are limited processes due to the fact that they are difficult to relate to actual processing conditions. Composites with a PHBV matrix filled with fibers of plant origin such as coconut [34,35,36,37,38], bamboo [39,40,41,42,43,44,45,46], abaca [47,48], pineapple [32,49] and sisal [50,51,52] have been the subject of research.

There is a noticeable amount of information on the possibility of processing and assessing the properties of PHBV composites with cellulose fibrous fillers such as flax, hemp and wood fiber—i.e., the most popular and available fibers in European countries. In this regard, for example, in the work of Keller [53], a PHBV composite was produced—hemp fiber by means of a co-rotating twin-screw extruder. Fibers with a length ranging from 5 to 25 mm were tested. PHBV with the trade name Biopol D400 GN was used as the polymer matrix. Due to the instability of filler introduction, manual dispensing of entangled fibers not only resulted in non-uniform granules but also caused fiber stagnation in the area of the die inlet, especially at higher fiber contents. In order to ensure stable work, an attempt was made, inter alia, to spinning to obtain a greater degree of homogenization, and the configuration of the screw zones was optimized to minimize damage to the fibers during the mixing process. Shortening of the fibers and loss of rectilinear geometry were noticed during the process. A composite with a volume fraction of fibers equal to 32% was obtained. The produced granulate was injected into the mold with the geometry of the samples intended for the uniaxial tensile test. The study also found that using hemp fibers in the PHBV matrix did not improve the tensile strength, while it was found that the maximum elongation was reduced compared to pure PHBV.

The work of Barkoul and co-authors [54] focused on the assessment of the properties of PHBV–flax fiber composites with variable flax fiber content. The tests were performed for samples produced by injection molding and compression molding. The polymer matrix was PHB with the trade name Biopol. Poly (3-hydroxyvaleric acid) was added to the PHB in an amount of 8 and 12% by weight. The length of the linen fibers used was approximately 10 mm, and the fibers arranged in the form of a mat 25 mm. The volume fraction of fibers in the polymer matrix was changed and amounted to: 0%, 20%, 30% and 40%. The mat saturated with biopolymer was pressed. The polymer matrix and the fibers were mixed using a rotary rheometer to prepare the injection molding granulate. The mixture was then granulated and injected into a mold. The mechanical properties of the obtained composites with the mass fraction of filler from 10% to 30% were compared. Similar values of Young’s modulus were noted for samples produced by the two methods. Slightly higher values of the elasticity modulus were obtained for injection molded samples (approximately 6 GPa at 30% by volume of the filler). In the case of the tensile strength and the maximum elongation of the samples, similar results were obtained for both methods. In the case of the Izod impact toughness test, significantly higher impact toughness values were obtained for pressed samples.

On the other hand, when analyzing the studies on the possibility of using short hemp, flax fibers in the PHBV matrix, little information was noticed regarding the production, processing and evaluation of the properties of this type of composites. The fibers used in the abovementioned works were usually characterized by quite a long length, with a very large statistical dispersion. This could be the reason for determining random properties of the obtained composites. In addition, fibers that were too long in relation to the diameter of the extruder screw and injection molding machine underwent mechanical degradation, as a result of which the said fiber length spread could be even greater. Moreover, there is no information on the modification of the surface of the fibers in order to improve the adhesion of the fiber to the matrix. The large and variable ratio of the fiber size to the size of the pure PHBV granulate also resulted in a very low degree of homogenization of the composites. Therefore, it was important to consider the possibility of using short fibers in the PHBV matrix, e.g., with a length of 1 mm and a very small length spread, and the matrix in the form of a powder. This would probably make it possible to obtain composites with a higher degree of homogenization. It should be mentioned that some of the works did not use conventional devices for the preparation of pellets, i.e., an extruder; hence, the observations and obtained results regarding the obtaining of composite pellets may not reflect possible phenomena and problems on an industrial scale. Based on the literature review on the composition and surface modification of plant fibers, it was noticed that the use of an appropriate method of fiber etching affects the properties of the obtained biocomposites. Therefore, research was undertaken on the influence of the surface treatment of flax and hemp fibers on the properties of the obtained PHBV matrix biocomposites.

## 2. Materials and Methods

### 2.1. Materials

The PHBV powder Enmat Y1000 trade name of Helian Polymers (Belfeld, The Netherlands) trade name was used as the polymer matrix. The molar proportion of HV in the biopolymer was 8%, the density of the biopolymer 1250 kg/m^3^ and the softening point ranged from 165 to 175 °C.

Hemp and flax fibers (delivered by EKOTEX company (Kowalowice, Poland)) with a length of approximately 1 mm and an average length-to-diameter ratio (L/d) of approximately 10 were used as the filler in the polymer matrix.

The produced biocomposites contained flax or hemp fibers with a 15% mass fraction. For the surface modification of the fibers, an aqueous sodium hydroxide solution with concentrations of 2%, 5% and 10% was used.

### 2.2. Sample Preparation

**Figure 1:**
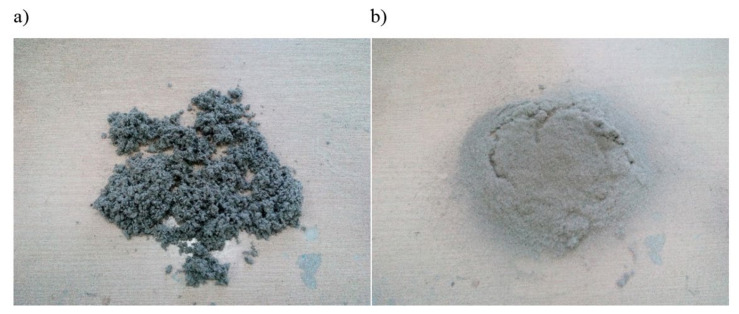
Hemp fibers alkalized with 10% NaOH solution: (**a**) before sieving, (**b**) after sieving.

The surface of the fibers was modified with sodium hydroxide solution for various concentrations, i.e., 2%, 5% and 10% NaOH. The selection of the percentage concentration was established based on the literature [55,56,57,58,59,60]. Mercerization was carried out for 1 h in a rotor device, whereby the fibers were constantly mixed with the solution. During the alkalization, the temperature of the mixture increased. Then, the fibers were washed with water until they were neutral, filtered off with a centrifuge and dried at 90 °C. As a result of the surface modification carried out, the fibers tended to form larger clusters (Figure 1a), which is an undesirable effect that could negatively affect the proper homogenization of the fiber–PHBV mixture. All clusters of fibers were screened on a sieve (the mesh size was 1.5 mm), thanks to which a more uniform distribution of filler size used in the PHBV was obtained (Figure 1b).

Eight types of PHBV matrix biocomposites with the following markings were produced in the extrusion process:

K0—containing hemp fibers not surface-modified;

K2—containing hemp fibers surface-modified with 2% NaOH solution;

K5—containing hemp fibers surface-modified with 5% NaOH solution;

K10—containing hemp fibers surface-modified with 10% NaOH solution;

L0—containing flax fibers not surface-modified;

L2—containing flax fibers surface-modified with 2% NaOH solution;

L5—containing flax fibers surface-modified with 5% NaOH solution;

L10—containing flax fibers surface-modified with 10% NaOH solution.

**Figure 2:**
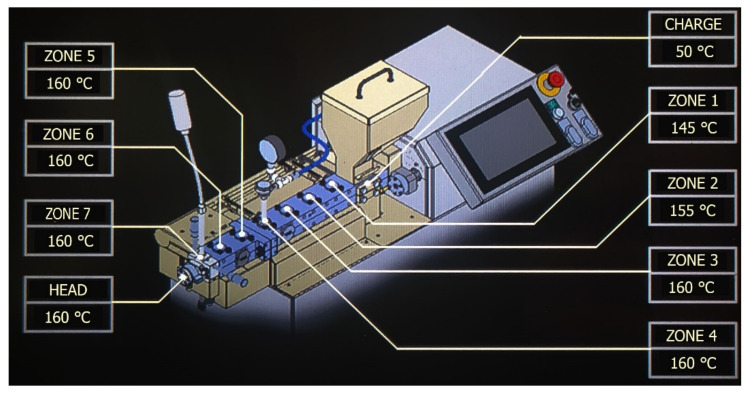
Areas on the individual heating zones of the twin screw extruder for K0 biocomposite extrusion.

The biocomposites were extruded using a ZAMAK REA-2P12A twin-screw extruder produced by ZAMAK Mercator company (Skawina, Poland) at constant temperature values on individual heating zones of the extruder in the range from 145 (zone 1) to 160 °C (head) (Figure 2). The list of set temperatures is presented in Table 1. Only in the case of biocomposite with flax fibers alkalized with 10% NaOH solution was it required to lower the temperatures by approximately 20 °C in all heating zones due to the very low viscosity of the extrudate obtained. At given default temperatures (such as during extrusion of other biocomposites), the extrudate was characterized by a very low viscosity, from which it was difficult to obtain extrudates of the correct length. Moreover, the extrusion process of this composite was unstable—jumps of the pressure value at the extruder head by approximately 30% were noticed.

Extrusion of all biocomposites was carried out with constant screw rotational speed of 50 rpm. Extrusion was carried out using an extrudate granulating station equipped with a cooling bath and a granulator. It should be noted that both the polymer matrix and the plant fibers were earlier dried for 3 h at a temperature of 90 °C before the extrusion process. The obtained granules were used for the production of specimens for testing mechanical properties by means of injection molding technology.

A DrBoy 55E injection molding maschine, produced by BOY Mashines Inc. (Exton, PA, USA) equipped with a Priamus system for monitoring and controlling the injection molding process was used for the manufacturing process of the samples.

An injection mold with inserts intended for uniaxial tensile testing (in accordance with PN-EN ISO 527-1) [61] was used in the tests. The “dog-bone” geometry samples for all types of biocomposites and biopolymers were manufactured with the adjustable parameters listed in Table 2. Only in the case of the biocomposite with flax fibers alkalized with 10% NaOH solution were the lower temperatures by approximately 20 °C in all heating zones of the injection molding machine required. At the set default temperatures, the injected material was characterized by a very low viscosity, which made it difficult to obtain a workpiece with the correct shape.

### 2.3. Methods

The Zwick Z030 testing machine was used to determine the strength properties of the obtained composites. The uniaxial tensile test was carried out in accordance with the EN ISO 527-1 standard for specimens with “dog-bone” geometry. Each series of specimens consisted of seven pieces for subsequent statistical analysis. On the basis of the obtained test results, the following were analyzed: Young’s modulus (E), tensile strength (σ_M_) and elongation at break (ε_M_). The results were statistically processed, where the arithmetic mean (AM), standard deviation (SD) and the coefficient of variation (CV) were determined. The Brinell method was used in accordance with PN-EN ISO 2039-1 [62] in two areas of samples intended for the uniaxial tensile test, i.e., in the measuring zone for the uniaxial tensile test (zone A) and in the gripping part (zone B). For this purpose, a Zwick 3106 hardness tester was used. Each series of samples consisted of seven pieces for subsequent statistical analysis.

Tests of biocomposite samples in the impact tensile test were carried out too. The impact tensile strength was determined in accordance with the EN ISO 8256 [63] standard. A CEAST 9050 pendulum hammer produced by Instron Inc. Europe (Buckinghamshire, UK) was used for this purpose. Sample geometry was modified according to the standard. The notch was milled for entire sample packages. Each series of samples consisted of seven pieces for subsequent statistical analysis.

The degree of water absorption of the produced samples was tested based on the EN ISO 62 [64] standard. The molding shrinkage of the “dog-bone” geometry was tested on the basis of the EN ISO 294-4 [65] standard.

The study of sample topography was carried out using a HITACHI S-3400 scanning electron microscope (SEM) produced by Hitachi Inc. (Tokyo, Japan). In order to visually assess the surface and geometry of the fibers and composites, a Nikon MM 800 workshop microscope produced by Nikon Inc. (Tokyo, Japan), a Nikon LV-100D optical microscope and a Alicona Infinite Focus 3D microscope produced by Alicona Imaging GmbH, (Raaba/Graz, Austria) were used.

## 3. Results

### 3.1. Assessment of the Surface Microstructure of Composites

In order to assess the degree of etching of plant fibers, a scanning electron microscopy study was carried out using a HITACHI S-3400 scanning electron microscope (SEM).

**Figure 3:**
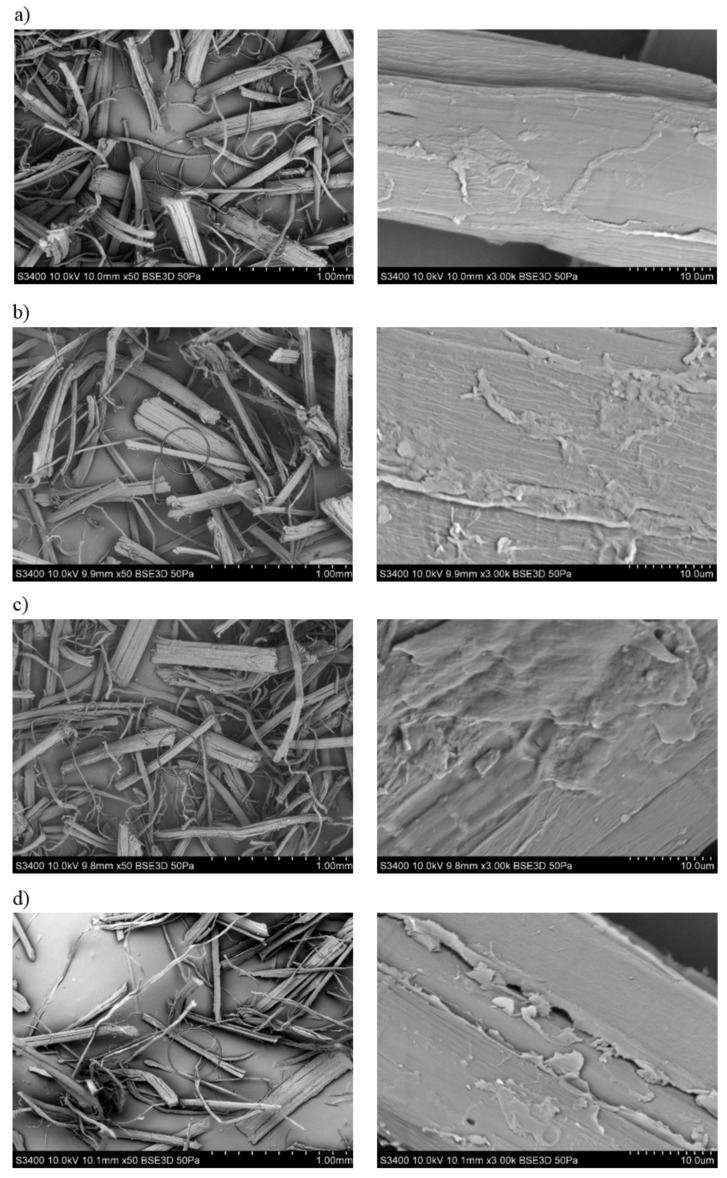
SEM photographs of hemp fibers: (**a**) unmodified, (**b**) exposed to 2% NAOH solution, (**c**) exposed to 5% NAOH solution, (**d**) exposed to 10% NAOH solution.

By analyzing the SEM photographs (Figure 3) of hemp fibers subjected to alkalization, it can be observed that the surface of the fibers is more developed than before the modification. Moreover, the fibers subjected to the mercerization process are characterized by a slightly smaller diameter, while maintaining a rectilinear geometry.

**Figure 4:**
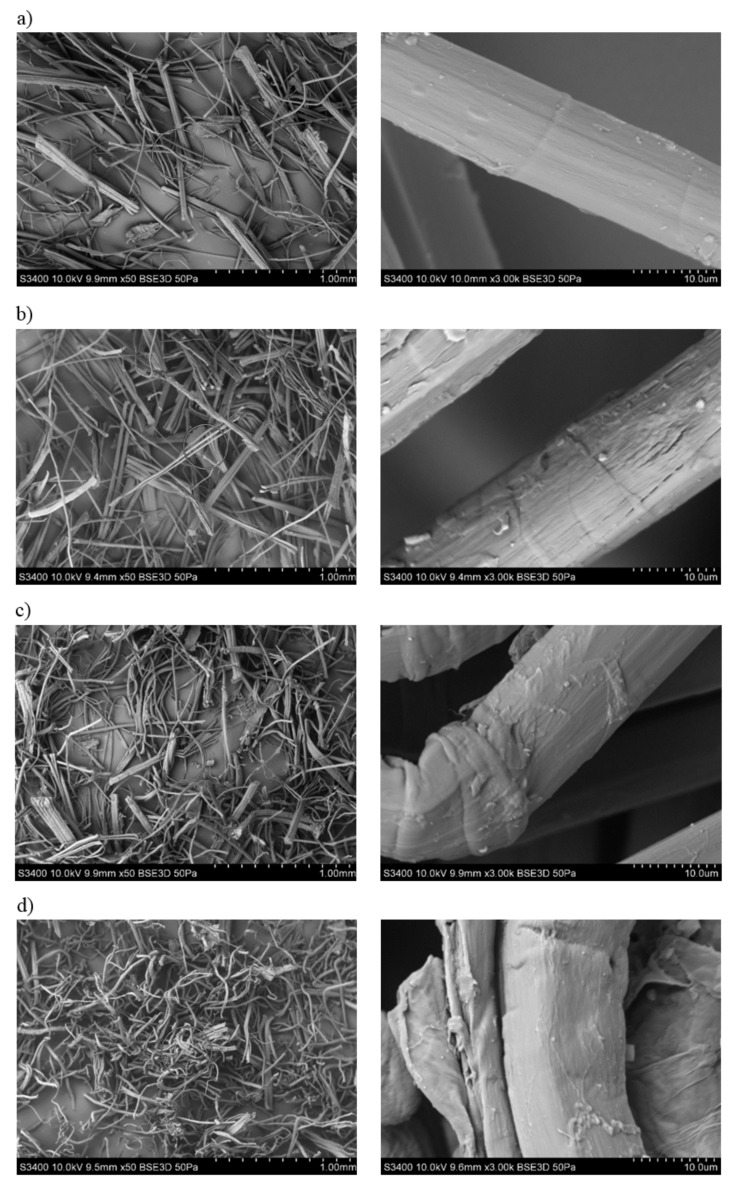
SEM photographs of flax fibers: (**a**) unmodified, (**b**) alkalized by 2% NAOH solution, (**c**) alkalized by 5% NAOH solution, (**d**) alkalized by 10% NAOH solution.

By analyzing the SEM images of flax fibers (Figure 4), an increase in the degree of surface development after treatment with NaOH solution can be observed. When treating the fibers with a 5% solution and especially with 10%, the fibers lose their rectilinear geometry; they are twisted and form groups of interconnected “bundles”. For all images of flax fibers after chemical modification, the diameter of the fibers is smaller.

### 3.2. The Fibers Shape Assessment of Factor Using Microscopic Examination

**Figure 5:**
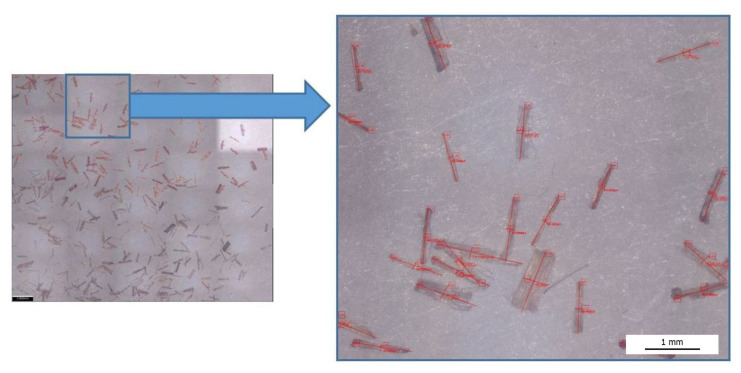
Example photograph of cellulose fibers for which the length and diameter were measured.

An analysis of the length and diameter of the fibers before and after surface modification was performed. For this purpose, photos of fibers were taken (Figure 5) using the Alicona Infinite Focus microscope. Then, after taking the photographs, about 100 fibers from each set were measured, where the length (L) and diameter (d) of each fiber were determined. The results were statistically analyzed (Table 3). The standard deviation (SD) (in relation to length—SD_L_, diameter—SD_d_) and the coefficient of variation (CV) (in relation to length—CV_L_, diameter—CV_d_) were determined.

When analyzing the results (Table 3) concerning the measurement of the length and diameter of the fibers, it should be noted that the diameter of flax fibers after modification using 10% NaOH solution was reduced by approximately 51%, which resulted in an increase in the shape factor to a value of approximately 21. On the other hand, the hemp fibers alkalized by 10% NaOH solution were characterized by a reduced diameter of approximately 18%, which resulted in the obtained shape factor of approximately 9. In the case of fiber length measurement, the degree of surface modification did not change the length value.

### 3.3. The Pressure Change Profile Analysis in the Mold Cavity

**Figure 6:**
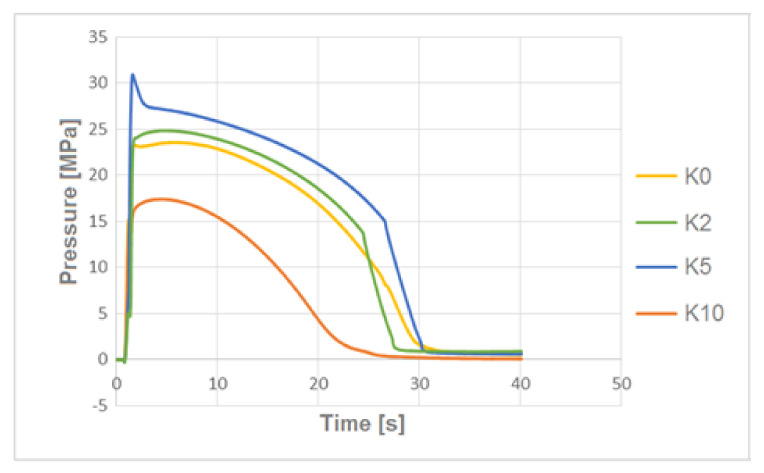
The pressure profiles in the mold cavity for composites filled with unmodified hemp fibers and modified with alkalized 2%, 5% and 10% NaOH solution.

**Figure 7:**
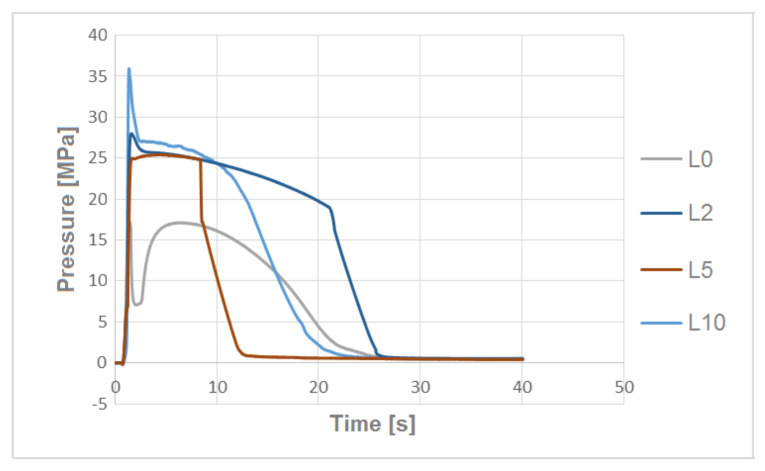
The pressure profiles in the mold cavity for composites filled with unmodified flax fibers and modified with alkalized 2%, 5% and 10% NaOH solution.

By analyzing the pressure profiles in the mold cavity for biocomposites filled with hemp fibers alkalized with various concentrations of NaOH solution (Figure 6), lower pressures in the mold cavity were obtained for the composite with fibers modified with 10% NaOH solution. In the case of pressure profiles for biocomposites filled with flax fibers (Figure 7), therewas a problem with obtaining the correct switching point during injection—this is evidenced by undesirable temporary pressure increase. Additionally, as the degree of flax fiber modification increased, higher pressure values were observed for the corresponding biocomposites.

The obtained samples were intended for testing the mechanical properties and quality of the molded piece.

### 3.4. The Surface Quality Assessment of the Molded Piece Using Optical Microscopy

**Figure 8:**
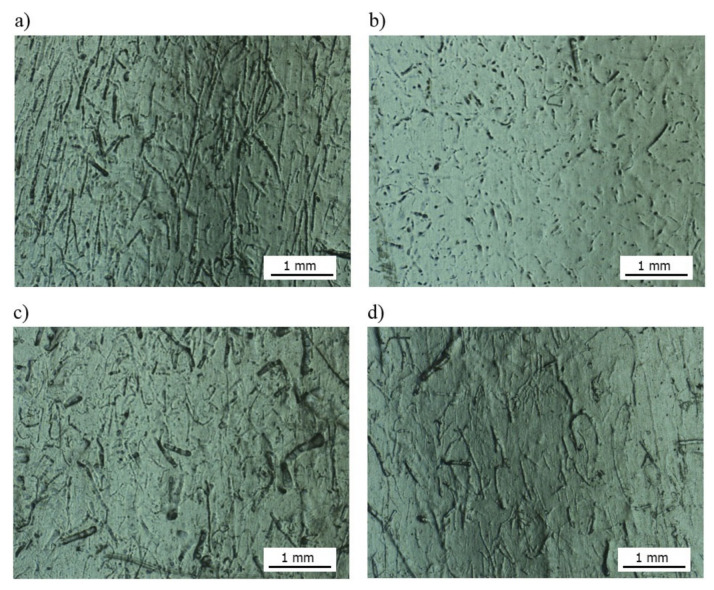
Areas of molded piece surface (50× magnification) for biocomposites: (**a**) L0, (**b**) L10, (**c**) K0, (**d**) K10.

Additional tests were carried out by means of an optical microscope to assess the quality of the sample surfaces of “dog-bone” shape (in the measuring zone) made of biocomposites filled with unmodified fibers and alkalized with 10% sodium hydroxide solution. It was observed (Figure 8) that the flax fibers after surface modification are barely visible in the polymer matrix, in contrast to unmodified flax fibers. This may indicate that the flax fibers modified with 10% NaOH solution are excessively etched, i.e., they degrade and therefore do not fulfill the proper filler function. In the case of hemp fibers modified with 10% NaOH solution, it was noticed that the degree of fiber dispersion is more favorable than that for a biocomposite with unmodified fiber—the fibers have a smaller diameter, are more regularly distributed in the polymer matrix and a greater unidirectional tendency is noticeable.

### 3.5. Determination of Mechanical Properties by Means of the Uniaxial Tensile Test

**Figure 9:**
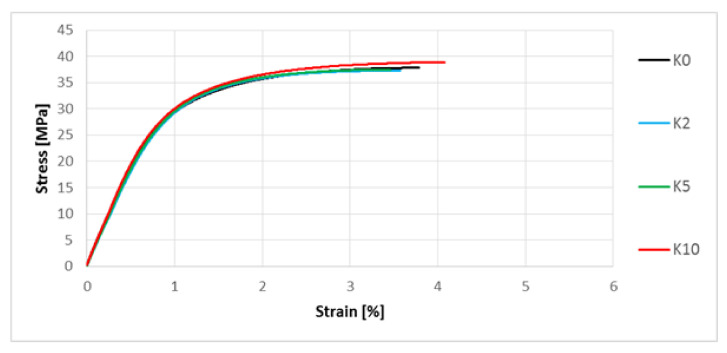
Stress–strain characteristics for composites with unmodified hemp fibers and those modified by 2%, 5% and 10% NaOH solution.

**Figure 10:**
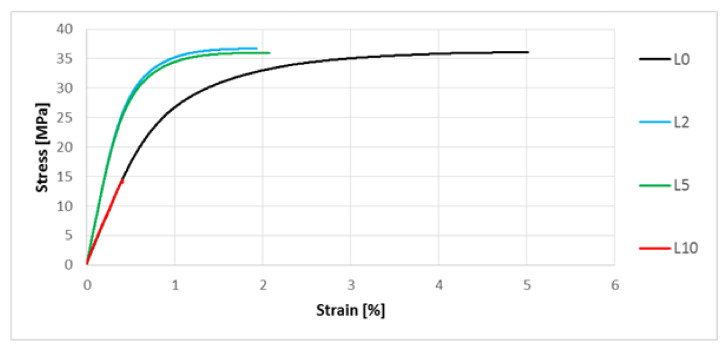
Stress–strain characteristics for composites with unmodified and modified flax fibers exposed to 2%, 5% and 10% NaOH solution.

A uniaxial tensile test was performed. The representative stress–strain characteristics are shown in Figure 9 and Figure 10.

Analyzing the results (Table 4), it was noticed that the alkalization on hemp fibers used as a filler in biocomposites had a positive effect on the increase of Young’s modulus in relation to the biocomposite with unmodified hemp fibers (increase by approximately 5% for the composite marked K10), as well as tensile strength (increase by approximately 1.5% for the composite marked K10). In the case of elongation at break, no direct relationship was found regarding the effect of examined solution concentration. In the case of biocomposites with fibers modified by 2% and 5% NaOH solution, there was a decrease in the elongation value, while for the biocomposite with fibers modified by 10% NaOH solution, the elongation value slightly increased compared to the biocomposite in which hemp fibers were not alkalized.

Analyzing the results (Table 5) of the static tensile test for composites containing flax fibers alkalized by 2% NaOH solution, an increase of the Young’s modulus (by approximately 10%) and tensile strength (by approximately 2%) was noted, as well as a of elongation at break (by approximately 17%) in relation to the biocomposite with unmodified flax fibers. In the case of a further increase in the concentration of the NaOH solution used on flax fibers, the mechanical properties of the biocomposites deteriorate, but they significantly decrease for the biocomposite with flax fibers modified with 10% NaOH solution. There was a decrease of elongation at break by about 91% and in tensile strength by about 58% compared to the composite with unmodified flax fibers.

### 3.6. The Shrinkage Biocomposites Assessment

**Figure 11:**
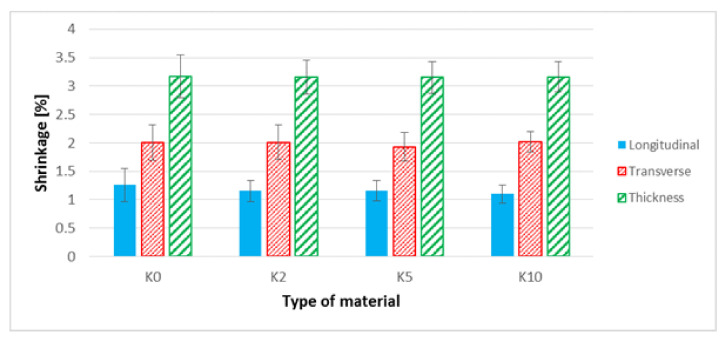
Linear shrinkage of samples: longitudinal, transverse and in thickness, for biocomposites filled with hemp fibers before and after surface modification.

Analyzing the results concerning the shrinkage of the samples (Figure 11), in the case of the hemp fiber modification by a 10% NaOH solution, the value of the longitudinal shrinkage of the biocomposite was reduced by approximately 13% compared to the samples made of the biocomposite filled with unmodified hemp fibers, but observed changes were lower than the error bar. Moreover, in the case of longitudinal contraction, a smaller scatter of the results for biocomposites with modified fibers was noted. In the case of transverse contraction of biocomposites filled with hemp fibers, no significant changes were observed after the fibers were alkalized by sodium hydroxide.

**Figure 12:**
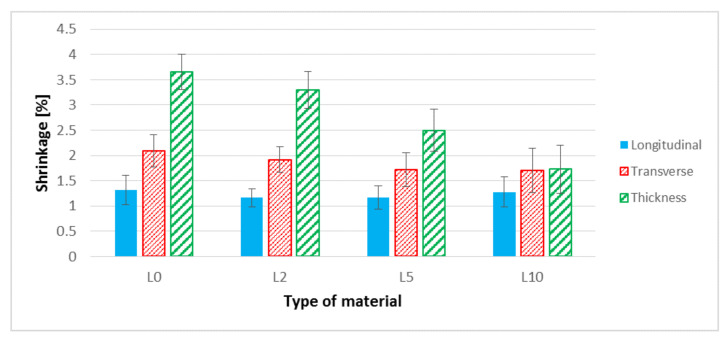
Linear shrinkage of samples: longitudinal, transverse and in thickness for biocomposites filled with flax fibers before and after surface modification.

Analyzing the results of the shrinkage (Figure 12) for samples made of biocomposites filled with flax fibers, a decrease in longitudinal shrinkage by approximately 12% for samples made of L2 and L5 biocomposites can be observed. In the case of transverse shrinkage and in thickness of the molded part, a significant decrease in the values for the L10 biocomposite was found. In the case of transverse shrinkage, this decrease was approximately 19% and for thickness shrinkage approximately 53% in relation to the biocomposite filled with unmodified flax fibers.

### 3.7. The Brinell Hardness Test

**Figure 13:**
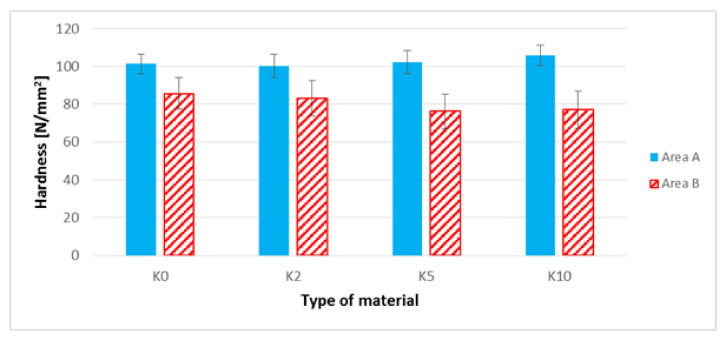
Hardness of composites filled with hemp fibers before and after surface modification in areas A and B of the sample.

**Figure 14:**
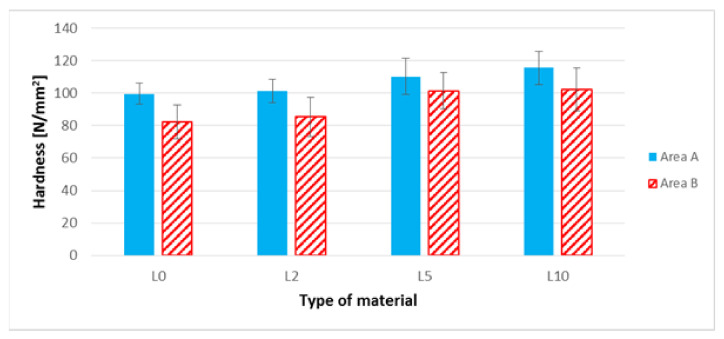
Hardness of composites filled with flax fibers before and after surface modification in areas A and B of the sample.

When interpreting the results of the hardness of biocomposites in area A (Figure 13 and Figure 14), an approximate 5% increase in the hardness value for the K10 biocomposite as compared to the K0 biocomposite can be observed. Also noticeable is an approximate 16% increase in the hardness value for L10 biocomposites in relation to the biocomposite filled with unmodified flax fibers. The highest hardness values in area A of all biocomposites discussed in the previous section was noted for the L10 biocomposite. When analyzing area B (Figure 13 and Figure 14), a slight decrease in the hardness value for biocomposites filled with modified hemp fibers can be noted.

In the case of biocomposites with flax fibers, an increase in the hardness of samples in area B was observed for biocomposites with modified flax fibers, modified with NaOH solution at a concentration of 5% and 10%. This increase was approximately 24% in relation to the biocomposite filled with unmodified flax fibers.

### 3.8. Impact Tensile Strength Test

**Figure 15:**
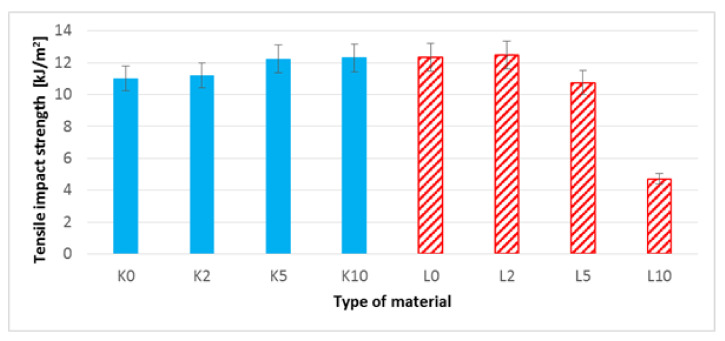
Impact tensile strength of biocomposites filled with hemp and flax fibers before and after surface modification.

When analyzing the results of the impact tensile strength (Figure 15), in the case of PHBV-hemp fiber biocomposites, an increase in the value of this parameter by approximately 12% (for K10) was noted in relation to the biocomposite with unmodified hemp fibers. On the other hand, in the group of biocomposites filled with flax fibers, a gradual decrease in impact tensile strength is visible along with the degree of etching of the fibers—a decrease in impact tensile strength can be noticed by a maximum of approximately 62% (for L10) compared to the biocomposite filled with unmodified flax fibers.

### 3.9. The Water Absorption Assessment

**Figure 16:**
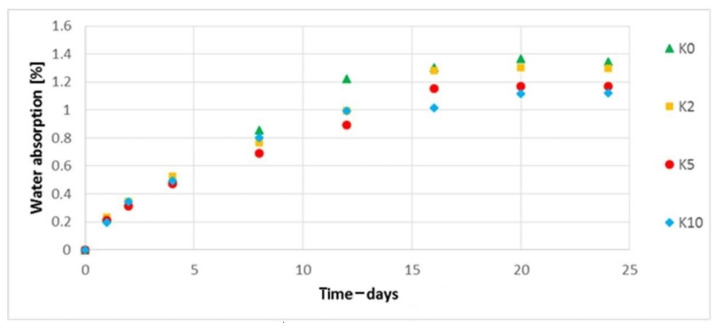
Water absorption of composites filled with unmodified and modified hemp fibers.

**Figure 17:**
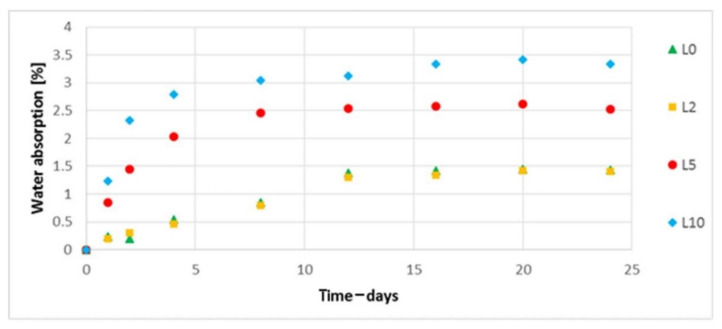
Water absorption of composites filled with unmodified and modified flax fibers.

The water absorption was assessed. In the case of biocomposites filled with hemp fibers (Figure 16), a decrease in the degree of water absorption was observed with an increase in the concentration of the alkalized solution used for alkalizing the fibers used in biocomposites. For the biocomposite filled with hemp fibers etched with 10% NaOH solution, an approximate 16% decrease in water absorption was noted (on the last day of the test). In the case of biocomposites filled with flax fibers (Figure 17), an increase in water absorption is visible for biocomposites with modified fibers (5% and 10% NaOH solution)—an approximate 133% maximum increase in water absorption was observed on the last day of the test (for L10) in relation to the biocomposite filled with unmodified fibers.

### 3.10. Assessment of the Composite Surface Microstructure

**Figure 18:**
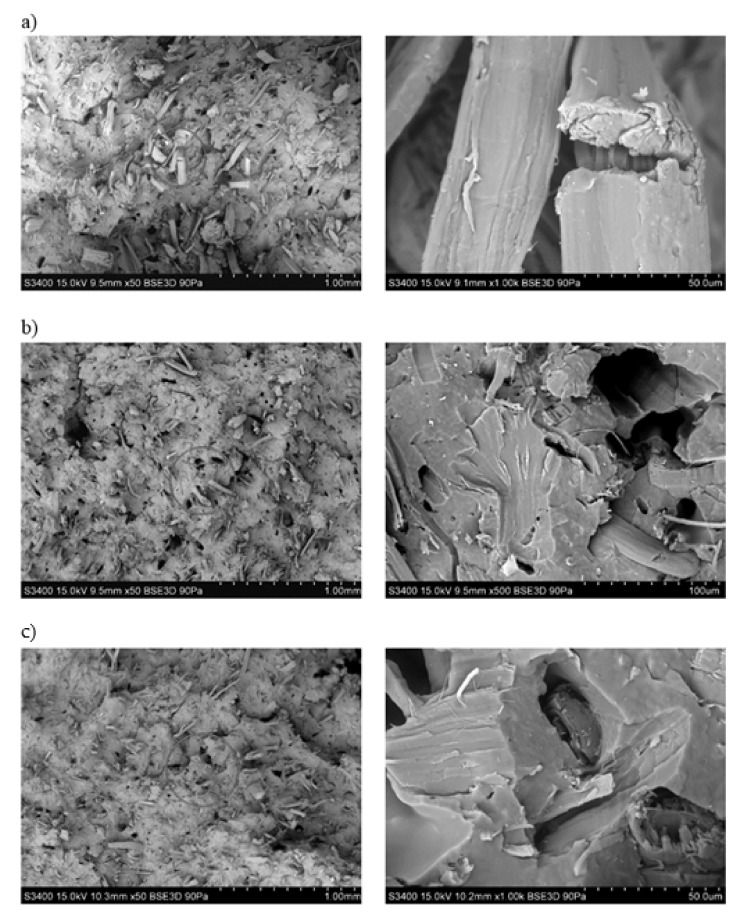
SEM photographs of fracture surface of biocomposite samples filled with hemp fibers: (**a**) unmodified, (**b**) alkalized with 2% NaOH solution, (**c**) alkalized with 10% NaOH solution.

**Figure 19:**
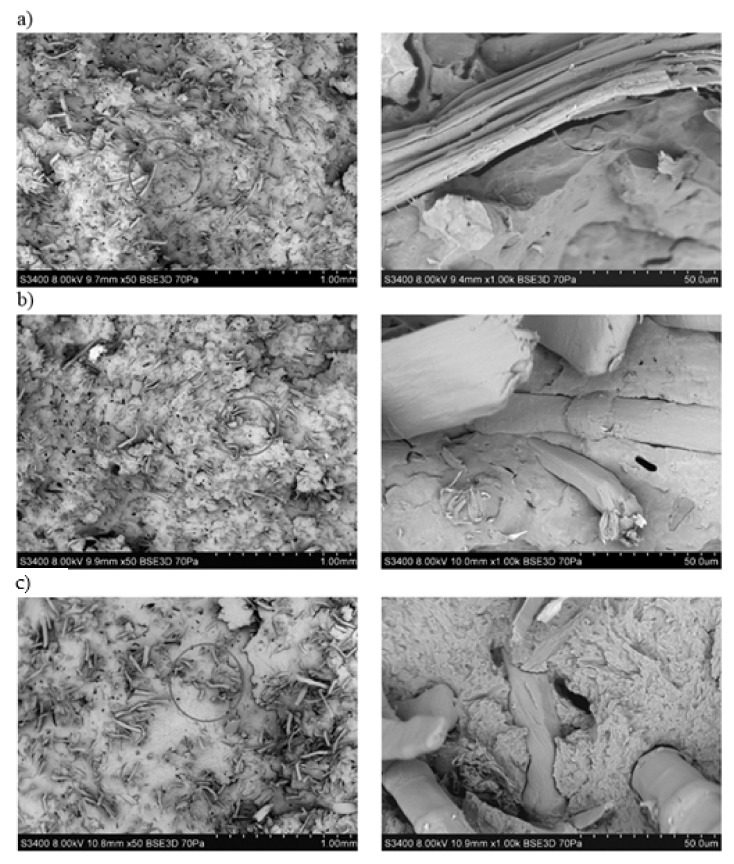
SEM photographs of fracture surface of biocomposite samples filled with flax fibers: (**a**) unmodified, (**b**) alkalized with 2% NaOH solution, (**c**) alkalized with 10% NaOH solution.

Analyzing the chosen SEM photos (Figure 18), which represent the fracture surface of specimens, it can be noticed that the hemp fibers after alkalization embedded in the polymer matrix have a reduced diameter. Moreover, no significant differences were observed in the microstructure of the biocomposites. In the case of biocomposites with flax fibers (Figure 19), as the alkalizing degree of fibers in the polymer matrix increases, the fibers become increasingly twisted and unevenly distributed in the matrix.

## 4. Discussion

The results presented in the paper cover four basic aspects: the structure of fibers and composites and the processing, mechanical and functional properties of biocomposites. Two types of fibers were alkalized with three NaOH solutions of different percentages. In the current known studies, the authors used different concentrations of the alkalizing solution for different types of fibers—there are no specific recommendations on what concentrations should be used to optimally improve the properties of the fibers. The percentages of NaOH solutions used in the study were determined based on the literature review. It should be emphasized, however, that in some studies 20% NaOH solutions were used, and the presented research focuses mainly on the topic of using a new, more widely unknown polymer matrix.

At the stage of producing biocomposites by extrusion, significant differences in the processing properties of the produced composites filled with various flax fibers were found. In the case of composites with fibers alkalized with 10% NaOH solution, it was noticed, by the same processing parameters, that the obtained biocomposite had a very low, unstable viscosity, making it impossible to use (with these technological parameters) this processing method. Reducing the processing temperature by 20 °C could stabilize the process and produce the correct extrudate. A similar phenomenon was also observed in the case of injection of test samples. In this case, the processing temperature was reduced by 20 °C (Table 2). The measured value of pressure in the mold cavity (Figure 7) for the L10 biocomposite was almost twice (pressure of 36 MPa) that of the composite with non-alkalized fibers (pressure of 17 MPa), which proves a significant increase in biocomposite viscosity (Figure 7).

In the case of composites with hemp fibers, pressure changes in the mold cavity followed a different trend (Figure 6)—for the composite, the fibers of which were etched with 10% NaOH solution, lower pressures in the cavity were recorded. This behavior may be due to the geometry of the fibers.

It was found that flax fibers significantly increased the aspect ratio L/d after being made alkaline with 10% NaOH solution. A particularly high value of L/d = 21.23 (Table 3) was observed for these fibers. The fibers were not shortened, and their diameter decreased by almost half, which may indicate a large delamination of the fibers in their structure. Moreover, when analyzing SEM pictures of flax fibers (Figure 4), not only was delamination visible but also a reduction in rectilinear geometry. Fibers became twisted (often even tangled), which proves a reduction in fiber stiffness.

In the case of the PHBV–flax fiber composite, the reduction of the L/d ratio for the fibers after etching with 10% NaOH solution (L10 composite) did not improve the mechanical properties of the composite. There was a decrease in the tensile strength (from 36.29 to 15.35 MPa), modulus of elasticity (from 3495.11 to 3352.45 MPa) and impact tensile strength (from 12.32 to 4.71 kJ/m^2^) compared to the composite, the fibers of which were not alkalized (L0). It was also found that the tensile strength deteriorated for composite L10 (15.45 MPa) as compared to pure PHBV (35.48 MPa) [66]. In addition, the observed change in the geometry of the fiber causes a decrease in the viscosity of the composite during extrusion and injection molding; hence, it was required to lower processing temperatures in both processes. These processing aspects are confirmed in the photographs of molded pieces made on the outer layer (Figure 8). Fibers of high stiffness, i.e., non-etched ones, constitute inclusions that act as an obstacle to the flow of the polymer matrix; hence, they are clearly visible on the outer layer of the molded pieces.

In the case of flax fibers etched with 10% NaOH solution, they are barely visible on the outer layer of the molded piece—as twisted bundles of low stiffness—preventing the flowing stream of polymer matrix; hence, they are immersed in the flowing core and slightly visible on the outer layer. In the case of composites with hemp fibers—both in the extrusion and injection molding processes—no significant differences were found, which is confirmed by the almost unchanged geometry of the fibers; despite being alkalized with 10% NaOH solution, they are still straight and stiff with slightly increased L/d aspect ratio (from 7.15 to 8.77), which indicates an almost slight reduction in the fiber diameter.

The next stage of the research focused on the assessment of selected mechanical properties. In the case of uniaxial tensile test results (Figure 9, Table 4) for composites with hemp fibers, a slight improvement in Young’s modulus and tensile strength were noted. This is most likely due to an increase in the L/d ratio and increased adhesion of the fibers to the polymer matrix. In the case of flax fibers, another relationship was noted—the L10 composite was characterized by the lowest values of Young’s modulus and tensile strength (Table 5, Figure 10), and the digested fibers, in the form of bundles of reduced stiffness, no longer fulfilled the function of a filler in the form of short fibers, ultimately not improving the mechanical properties in the direction of force. In the case of hardness, for composites with hemp fibers (Figure 13), a difference in hardness was noted in the tested measurement zones; in the grip part of the tested molded part, the hardness was lower than in the measuring part of the sample. This difference is due to the fact that during the filling of the mold cavity in the injection process, a change in the geometry of the mold cavity in the nip part results in the disorientation of the fibers, i.e., their more chaotic arrangement. On the other hand, in the measuring part of the dog-bone sample, the stream of polymer with the fiber flows for a longer time in the channel with a constant cross-section, which results in unidirectional arrangement of the fibers of this part of the mold cavity. As can be seen, the increase in the hardness of the composite is slight after the surface modification of the hemp fibers. In the case of composites with flax fibers (Figure 14), a completely different trend was noted—the fibers no longer function as a typical fibrous filler, and their chaotic arrangement as coiled bundles of low stiffness does not result in the formation of resistance during the polymer flow, which in turn translates into deep fibers sinking into the internal layers of the tested specimen. This is confirmed by very small differences in hardness in the measurement zones of specimens. On the other hand, when analyzing the hardness results in terms of fiber alkalization, an upward trend was noted with increasing concentration of the alkalizing solution. Flax fibers, increasingly digested, twisted and less stiff, settle in the core of the molded part, and due to the lowering of the viscosity of the flowing plastic stream, the mold cavity is “more tightly packed”, which translates into an increase in the density of the entire material. This also translates into an improvement in thickness shrinkage. Impact tensile strength results for hemp fiber composites show the classic trend of improved adhesion of the fibers to the matrix and increased L/d ratio.

When analyzing the performance parameters of the obtained composites, it is necessary to mention the changes in shape and dimensions. In the case of shrinkage of the molded piece, a slight (within the measuring error) reduction of the longitudinal shrinkage was observed. This is probably due to a slight increase of the fiber aspect ratio. In turn, in the case of flax fibers, the previously described trend can be seen with increasing NaOH concentration. The straightness and stiffness of the fibers decrease, which reduces their role as a fibrous filler, which does not change the longitudinal shrinkage. On the other hand, a significant reduction in thickness shrinkage is the result of major “packing” the mold cavity with a composite of lower viscosity. Such “packing” of the cavity is the cause of increased water absorption in the case of the composite with fibers etched with 10% NaOH solution. Taking into account the SEM photographs of the molded piece (Figure 8b), the fibers are less deposited on the outer layer; hence, the tendency to absorb water should be lower. On the other hand, in the case of composites with hemp fibers, the water absorption decreases with the increase of NaOH concentration; fibers have a more developed surface, increasing the adhesion of the fibers to the matrix.

The research carried out in this work is therefore a novelty in the context of production, processing and also in the evaluation of the properties of the obtained composites in terms of processing, usability and evaluation of mechanical properties. Moreover, the paper shows the methods of preparing flax and hemp fibers in terms of the desired properties of the products.

## 5. Conclusions

The obtained test results indicate that the effect of the alkalizing solution concentration should be selected individually for a specific type of natural fiber and polymer matrix.

The alkali treatment of cellulose fibers causes significant changes of the fiber diameters; it is especially visible for flax fibers, where the diameter reduction was over 50% compared to unmodified fibers (L0 composite) to fibers modified by 10% of NaOH solution (L10 composite).

Slight improvement of Young’s modulus, tensile strength and hardness of composites with hemp fibers alkalized by NaOH solutions was found, and the best results were observed for biocomposites filled with hemp fibers modified with 10% NaOH solution. In addition, in terms of processing (for the same processing parameters), lower (by about 47%) cavity pressures were reported for the same biocomposite with fibers alkalized by means of 10% NaOH solution.

When the concentration of the alkalizing solution for flax fibers increases, no analogous relationship can be seen. It can be noticed that the fibers are quite sensitive to the action of sodium hydroxide; after treating the fibers with a 2% NaOH solution, most of the discussed properties (Young’s modulus, tensile strength, shrinkage of samples) of the biocomposite slightly improved. On the other hand, after using higher concentrations of NaOH solutions, most of the properties of biocomposites filled with flax fibers deteriorated. This is especially visible for samples filled with flax fibers alkalized with 10% NaOH solution, where the mechanical properties of the biocomposite designated by the uniaxial tensile test deteriorated significantly as well as in the impact tensile strength test, and the degree of water absorption increased. Processing (extrusion and injection molding) of a biocomposite, the flax fibers of which were alkalized with 10% NaOH solution, was unstable—higher pressure values with greater fluctuations were achieved. In the SEM tests it was found that the flax fibers alone and embedded in the matrix after the surface modification with 10% NaOH solution twist and form larger groups. This may indicate the fiber diameter reduction and loss of properties as a fibrous filler.

The research presented in the paper show that the use of a 10% NaOH solution on hemp fibers improves the properties of the biocomposite in most of the analyzed areas. When using flax fibers, it is difficult to indicate the correct concentration of the alkalizing solution. To improve the Young’s modulus and tensile strength, 2% NaOH should be used. On the other hand, when using 10% NaOH, shrinkages of the molded piece (L10) decreased (reduced by approximate maximum values of longitudinal 9%, transverse 21%, thickness 61%) and hardness increased significantly (by an approximate maximum of 24%) compared to unmodified fibers (for L0 composite). The results obtained for the L10 composite are puzzling; hence, there is a great need to understand the causes of this phenomenon. For this purpose, it is planned to perform, inter alia, DSC and TGA tests as well as to determine the composite processing window and to explain phenomena described in this article.

Moreover, the results presented in the article constitute the basis for further research directions aimed at examining other methods of surface modification of fibers used as a filler in the PHBV matrix.

## Figures and Tables

**Table 1:** The temperatures set on the individual heating zones of the twin screw extruder.

Type of Biocomposite	Head	Zones 3–7	Zone 2	Zone 1	Charge
K0, K2, K5, K10, L0, L2, L5	160 °C	160 °C	155 °C	145 °C	50 °C
L10	140 °C	140 °C	135 °C	125 °C	50 °C

**Table 2:** The processing parameters of samples for uniaxial tensile testing.

Parameter	K0, K2, K5, K10, L0, L2, L5	L10
Mold temperature (°C)	60	60
Melt temperature (°C)	167	147
Cooling time (s)	25	25
Packing time (s)	25	25
Packing pressure (MPa)	30	30
Flow rate (cm3/s)	35	35
Mold temperature (°C)	60	60

**Table 3:** Average value of hemp and flax fiber length and diameter as well as the shape factor in statistical analysis.

Type of Biocomposite	L (mm)	SD_L_	CV_L_	d (mm)	SD_d_	CV_d_	L/d
L0	1.002	0.114	11.38	0.095	0.058	61.05	10.55
L2	0.994	0.113	11.37	0.090	0.055	61.11	11.04
L5	1.001	0.118	11.79	0.065	0.038	58.46	15.40
L10	0.998	0.121	12.12	0.047	0.029	61.70	21.23
K0	0.987	0.112	11.35	0.138	0.051	36.96	7.15
K2	1.003	0.102	10.17	0.134	0.050	37.31	7.49
K5	0.997	0.099	9.93	0.121	0.038	31.40	8.24
K10	0.991	0.095	9.59	0.113	0.042	37.17	8.77

**Table 4:** Results from the uniaxial tensile test for biocomposites with unmodified hemp fibers and fibers exposed to 2%, 5% and 10% NaOH solution.

Type of Biocomposite	Statistics	E (MPa)	σ_M_ (MPa)	ε_M_ (%)
K0	AM	3815.78	37.58	3.83
	SD	24.00	0.23	0.06
	CV	0.63	0.62	1.66
K2	AM	3855.64	37.17	3.57
	SD	45.64	0.84	0.07
	CV	1.18	2.25	2.02
K5	AM	3885.91	37.65	3.73
	SD	47.73	0.52	0.08
	CV	1.23	1.42	2.32
K10	AM	3992.55	38.11	4.05
	SD	43.10	1.08	0.14
	CV	1.12	2.84	3.45

**Table 5:** Results from the uniaxial tensile test for biocomposites with unmodified flax fibers and exposed to: 2%, 5% and 10% NaOH solution.

Type of Material	Statistics	E (MPa)	σ_M_ (MPa)	ε_M_ (%)
L0	AM	3495.11	36.29	5.07
	SD	41.71	0.53	0.25
	CV	1.19	1.45	4.90
L2	AM	3860.63	36.94	4.21
	SD	45.22	0.34	0.12
	CV	1.17	0.98	3.38
L5	AM	3762.51	35.78	4.00
	SD	55.10	0.82	0.07
	CV	1.46	2.28	1.81
L10	AM	3352.45	15.35	0.46
	SD	143.21	1.29	0.07
	CV	4.27	8.42	16.09

## Data Availability

The data presented in this study are available on request from the corresponding author.
